# A Pilot Study Evaluating Associations between Continuous Glucose Monitoring Metrics in Pregnancy and Postpartum A1c and Blood Pressure

**DOI:** 10.1055/a-2837-6898

**Published:** 2026-03-30

**Authors:** Chloe F. Michalopoulos, Diana C. Soria-Contreras, Sarah Hsu, Robin Azevedo, Arantxa Medina Baez, Emily A. Rosenberg, Camille E. Powe

**Affiliations:** 1 Diabetes Unit, Endocrine Division, Massachusetts General Hospital, Boston, Massachusetts, United States; 2 Division of Endocrinology, Diabetes and Metabolic Diseases, Medical University of South Carolina, Charleston, South Carolina, United States; 3 Department of Obstetrics and Gynecology, Massachusetts General Hospital, Boston, Massachusetts, United States; 4 Harvard Medical School, Boston, Massachusetts, United States

**Keywords:** pilot and feasibility study, diabetes risk assessment, gestational diabetes, continuous glucose monitoring, pregnancy

## Abstract

**Objective:**

This study aimed to examine the relationship between continuous glucose monitoring (CGM) metrics during gestational diabetes mellitus (GDM)-affected pregnancy and postpartum cardiometabolic measures in a pilot study.

**Study Design:**

We enrolled participants >6 months postpartum from a previous GDM trial where they wore a CGM during the third trimester. At the postpartum visit, we assessed hemoglobin A1c (HbA1c) and blood pressure (BP). We used linear regression models adjusted for age and body mass index (BMI) at the time of CGM wear to test for a relationship between pregnancy CGM metrics (mean glucose, coefficient of variation, time in pregnancy range 63–140 mg/dL [pTIR], time >120 and >140 mg/dL) and postpartum outcomes (HbA1c and BP).

**Results:**

Of 14 eligible participants with pregnancy CGM data, 11 (79%) returned at a mean of 20.3 months postpartum (range 11–33). Age and BMI during pregnancy CGM wear were 36.0 (2.7) years and 28.7 (5.5) kg/m ^2^ ; gestational age was 32.0 (2.0) weeks. Higher pTIR was associated with lower postpartum HbA1c ( *n* = 8, β = −0.06, *p* = 0.007). Other CGM metrics were not associated with HbA1c. There were no associations between CGM metrics and BP.

**Conclusion:**

Third-trimester CGM pTIR should be tested as a predictor of postpartum glycemia in a larger study.

## Introduction

Type 2 diabetes (T2D) and its precursor, prediabetes, affect over 100 million U.S. adults, including more than 25% of those of childbearing age. 1 Pregnancy offers a critical window for early detection and prevention of future T2D and cardiovascular disease (CVD) through universal screening for gestational diabetes mellitus (GDM) at 24 to 28 weeks' gestation. 2 3 4

After GDM, current guidelines recommend an oral glucose tolerance test (OGTT) at 4 to 12 weeks postpartum for diabetes risk assessment, yet completion rates for this test are often below 50%. 5 6 This limits the identification of individuals who could benefit from targeted prevention. Beyond diabetes, individuals with GDM are at elevated risk for hypertension and long-term CVD, 7 8 9 underscoring the need for a more accessible approach to chronic disease prevention.

Continuous glucose monitoring (CGM), now U.S. Food and Drug Administration (FDA)-cleared for use during pregnancy, offers a promising alternative for future diabetes risk assessment. These wearable sensors track glucose levels every few minutes and are increasingly used in GDM management. 10 11 If CGM data from pregnancy can predict future glycemic and cardiovascular risk, it could replace the low-yield postpartum OGTT and enable earlier, personalized interventions.

This pilot and feasibility study aimed to examine the relationship between CGM metrics during the third trimester of pregnancy and hemoglobin A1c (HbA1c) levels more than 6 months after a pregnancy affected by GDM. Secondary goals include assessing CGM metric associations with blood pressure (BP) and evaluating the feasibility of recalling participants from a prior pregnancy CGM study for follow-up. The pilot was designed to inform future studies focused on predicting and preventing both T2D and CVD in this high-risk population.

## Methods

### Population

Participants were recruited from a previous pregnancy study called Heterogeneity Informed Nutrition Therapy for Gestational Diabetes Mellitus (HINT-GDM). 12 Briefly, HINT-GDM was a randomized, crossover pilot study aimed at evaluating the impact of dietary macronutrients on postprandial glucose in people with diet-controlled GDM. During the third trimester of pregnancy, HINT-GDM participants attended a study visit where a CGM was placed and worn for at least 7 days. They were then asked to eat two different study breakfast meals in a randomized sequence (high-fat/low-protein or high-protein/low-fat). The participants consumed the test meals at home on consecutive mornings after fasting, while CGMs tracked post-meal glucose responses. Sixteen HINT-GDM participants had adequate pregnancy CGM data for analysis. From this group, we invited those who were more than 6 months postpartum to this follow-up study, HINT-Postpartum.

HINT-Postpartum consisted of a single in-person study visit in which we assessed weight, HbA1c, and BP, and collected general health history information such as medication use and medical diagnoses via participant interview. Weight was measured twice with a calibrated scale and averaged. HbA1c was measured via Roche Turbidimetric Inhibition Immunoassay in the Massachusetts General Hospital core clinical laboratory. Systolic and diastolic BP were each measured twice manually with a standard sphygmomanometer, and the average was reported.

The exposures were third-trimester CGM metrics (7-day wear period), including mean glucose (mg/dL), percent time in pregnancy range (pTIR; 63–140 mg/dL), percent time above 140 mg/dL, percent time above 120 mg/dL, and glucose variability assessed by the coefficient of variation (CV). Cardiometabolic outcomes included postpartum HbA1c (%, primary outcome) and systolic and diastolic BP (mm Hg, secondary outcomes).

### Statistical Analysis

Descriptive statistics were used to summarize participant characteristics. Continuous variables are presented as mean (standard deviation [SD]), and categorical variables as frequencies and percentages.

We examined the associations between third-trimester CGM metrics and postpartum cardiometabolic outcomes using Pearson correlation coefficients and linear regression models adjusted for age and body mass index (BMI) at the HINT-GDM pregnancy visit. For analyses of postpartum HbA1c, we excluded two individuals who were using glucose-lowering medications at the time of their postpartum assessment. After running linear regression diagnostics on the HbA1c models, an influential outlier (based on leverage, Cook's D, D-fit) 13 was excluded from subsequent HbA1c analyses.

We conducted two sensitivity analyses, one excluding a participant who experienced an intervening pregnancy between the HINT-GDM pregnancy and postpartum assessment. The second analysis further adjusted models for the number of months elapsed since delivery to account for the potential influence of time since pregnancy.

## Results

**Fig. 1:**
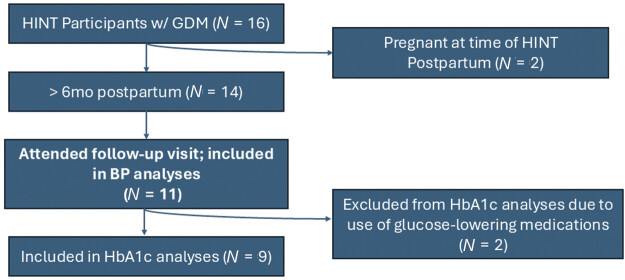
HINT postpartum study flowchart. Participants from the HINT-GDM study who were at least 6 months postpartum and not pregnant were invited to join HINT-Postpartum. About 79% of invited participants enrolled in HINT-Postpartum. Two participants were excluded from HbA1c analyses due to the use of glucose-lowering medications. An additional participant was excluded after regression diagnostics due to being an influential outlier. GDM, gestational diabetes mellitus; HbA1c, hemoglobin A1c; HINT-GDM, Heterogeneity Informed Nutrition Therapy for Gestational Diabetes Mellitus.

Of 16 participants who previously had GDM and participated in HINT-GDM, 14 were eligible for HINT-Postpartum (not pregnant at the time of follow-up), and 11 participants attended the HINT-Postpartum follow-up visit ( Fig. 1 ). These participants had a mean (SD) age of 36 (2.7) years, BMI of 28.7 (5.5) kg/m ^2^ , and gestational age of 32.0 (2.0) weeks at the HINT-GDM pregnancy visit. CGM summary metrics from the HINT pregnancy visit are shown in Table 1 . Participants attended the postpartum visit at a mean of 20.3 (range 11–33) months after delivery ( Table 1 ). At the postpartum visit, the mean (SD) BMI was 26.2 (4.8) kg/m ^2^ ; HbA1c was 5.4 (0.3)%; systolic BP was 103.4 (12.3) mm Hg; and diastolic BP was 68.5 (10.2) mm Hg.

**Table 1:** Participant characteristics

Characteristic	Mean (SD) or median [IQR] *N* = 11
**Pregnancy measures**	
Age a , years	36.0 (2.7)
BMI a , kg/m ^2^	28.7 (5.5)
Gestational age a , weeks	32.0 (2.0)
**CGM metrics**	
Mean glucose, mg/dL	83.7 (10.9)
Glucose CV	22.1 (3.3)
Percentage time >120 mg/dL	3.5 [1.8–4.9]
Percentage time >140 mg/dL	0.8 [0.6–1.1]
Percentage time in pregnancy range	87.7 (4.0)
**Postpartum measures**	
BMI b , kg/m ^2^	26.2 (4.8)
HbA1c, %	5.4 (0.3)
SBP, mm Hg	103.4 (12.3)
DBP, mm Hg	68.5 (10.2)
Months postpartum since last pregnancy c	20.3 (6.9)

Abbreviations: BMI, body mass index; CV, coefficient of variation; DBP, diastolic blood pressure; HbA1c, hemoglobin A1c; IQR, interquartile range; SBP, systolic blood pressure; SD, standard deviation.

a Age, BMI, and gestational age during HINT pregnancy at interventional visit.

b *N* = 10 for postpartum BMI due to one participant with missing weight data at HINT-Postpartum visit.

c Last pregnancy was the Heterogeneity Informed Nutrition Therapy for Gestational Diabetes Mellitus (HINT-GDM) pregnancy for everyone, with the exception of one participant with an intervening pregnancy. The range of postpartum visit attendance was 11 to 33 months.

**Fig. 2:**
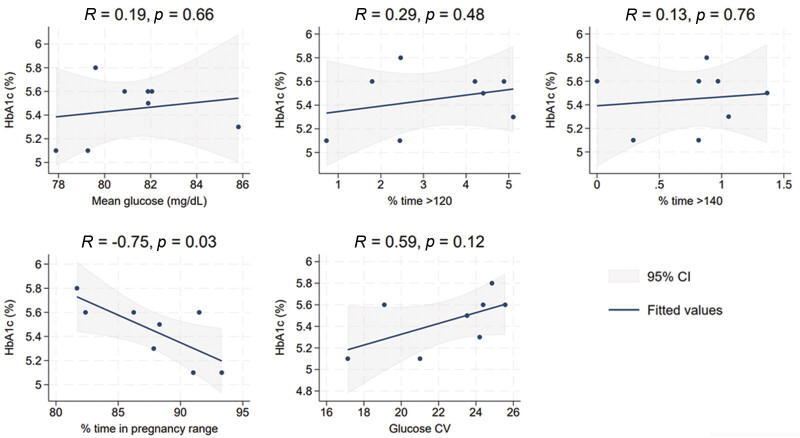
Pregnancy CGM metrics versus postpartum HbA1c. Shown are correlation coefficients between pregnancy CGM metrics and HbA1c at HINT-Postpartum visit ( *N* = 8). An influential outlier was excluded based on linear regression diagnostics. CGM, continuous glucose monitoring; CV, coefficient of variation; HbA1c, hemoglobin A1c.

When we assessed the correlations between CGM metrics and cardiometabolic outcomes ( Fig. 2 ), higher pTIR was correlated with lower postpartum HbA1c ( *r* = −0.75, *p* = 0.03). Other CGM metrics were not correlated with postpartum HbA1c ( Fig. 2 ). In BMI- and age-adjusted linear regression models, higher pTIR was associated with lower HbA1c (β = −0.06%, *p* = 0.007, Table 2 ). Mean CGM glucose, glucose CV, percent time above 120 mg/dL, and above 140 mg/dL were not associated with postpartum HbA1c ( Table 2 ) in adjusted models. Results including the influential outlier are shown in Supplementary Table S1 (available in the online version only). There were no associations between CGM metrics and postpartum systolic and diastolic BP ( Table 3 ).

**Table 2:** Age and body mass index-adjusted linear regression models for continuous glucose monitoring metrics and postpartum hemoglobin A1c a

Exposure	β coefficient	*p* -Value
Mean glucose	0.03	0.71
pTIR	−0.06	0.007
Percent time >140 mg/dL	−0.11	0.78
Percent time >120 mg/dL	0.07	0.41
Glucose CV	0.06	0.11

Abbreviations: CV, coefficient of variation; pTIR, percent time in pregnancy range.

a In HbA1c models, two participants on glucose-lowering medication and an influential outlier were excluded ( *N* = 8). Results, including outliers, are given in Supplementary Table S1 (available in the online version only).

**Table 3:** Age and body mass index-adjusted linear regression models for continuous glucose monitoring metrics and postpartum blood pressure a

Exposure	BP measure	β coefficient	*p* -Value
Mean glucose	Systolic BP	−0.48	0.28
	Diastolic BP	−0.46	0.14
pTIR	Systolic BP	−0.89	0.44
	Diastolic BP	−0.56	0.50
Percent time >140 mg/dL	Systolic BP	−1.29	0.35
	Diastolic BP	−1.35	0.15
Percent time >120 mg/dL	Systolic BP	−0.45	0.38
	Diastolic BP	−0.47	0.19
Glucose CV	Systolic BP	0.97	0.49
	Diastolic BP	0.65	0.51

Abbreviations: BP, blood pressure; CV, coefficient of variation; pTIR, percent time in pregnancy range.

a In BP models, all participants were included in analyses ( *N* = 11).

Findings were consistent with the primary analyses when we excluded a participant with an intervening pregnancy and accounted for the time elapsed since delivery in sensitivity analyses (data not shown).

## Discussion

In this pilot study, we examined the relationship between CGM metrics during a GDM-affected pregnancy and postpartum HbA1c and BP. Our data suggest that, among individuals with diet-controlled GDM, a longer time in pregnancy range in the third trimester is associated with lower HbA1c at an average of 20 months after delivery. No other CGM metrics were associated with HbA1c, and no metrics were associated with BP, though we cannot rule out that null findings were due to limited statistical power. Seventy-nine percent of eligible participants returned for the follow-up visit, suggesting the feasibility of longitudinal data collection.

Our findings extend previous large cohort studies demonstrating GDM is associated with future T2D risk. 4 14 15 Current clinical strategies require a postpartum OGTT prior to intervening for diabetes prevention in people with a history of GDM. Yet, postpartum OGTT completion rates are poor. 6 16 17 This highlights the need for alternative strategies to risk-stratify people with GDM that do not rely on postpartum testing. Recent studies have found that early (1–2 days) postpartum glycemic testing prior to discharge from the delivery hospitalization has similar predictive value to a 6- to 12-week postpartum OGTT for diabetes at 1 year postpartum in women with recent GDM. Further completion rates for early postpartum OGTT were >95%. 6 16 17

Our findings indicate that risk stratification may be possible even earlier: During pregnancy. CGM data, specifically pTIR, are associated with HbA1c at an average of 20 months postpartum, presenting a novel approach. Pregnancy is a time period when people are highly engaged with health care and willing to make lifestyle changes. 18 19 20 With the increasing use of CGM for GDM management, our studies suggest a potential opportunity to replace onerous postpartum testing with easily obtainable CGM metrics from pregnancy, though this needs to be tested in further, larger studies.

Although studies suggest that GDM can also be predictive of postpartum hypertension and CVD, 9 21 we did not observe any associations between CGM metrics and postpartum BP. This could be due to the limited sample size. Further, well-powered studies should study the relationship between CGM metrics in pregnancy and postpartum cardiovascular outcomes in GDM.

There are limited studies with available CGM data during pregnancy to use for postpartum outcome analysis. We were unable to identify prior studies that have examined the relationship between pregnancy CGM metrics and postpartum cardiometabolic outcomes. Limitations of our study include its sample size and the fact that CGM during pregnancy was available for only a 7-day wear period.

In conclusion, in this small pilot and feasibility study, we found an association between higher CGM pregnancy-specific time in range (63–140 mg/dL) in the third trimester and lower HbA1c at an average of 2 years postpartum. Our findings should prompt larger prospective studies to robustly test the relationships between pregnancy CGM metrics and the development of future prediabetes or diabetes.
