# Supplementary material for: A Pilot Study Evaluating Associations between Continuous Glucose Monitoring Metrics in Pregnancy and Postpartum A1c and Blood Pressure
Source: AJP Rep. 2026 Mar 30;16(1):e72–6. doi: 10.1055/a-2837-6898 (PMC13035414; doi:10.1055/a-2837-6898)
Supplement: Supplementary file 1 — Supplementary Material [file 10-1055-a-2837-6898_28459339.pdf]

Supplementary Table S1. Age and BMI-adjusted linear regression models for CGM metrics and postpartum HbA1c including influential outlier.

**Exposure**	**β Coefficient**	**P-value**
Mean glucose	-0.02	0.15
pTIR	-0.05	0.12
% time >140 mg/dl	-0.05	0.12
% time >120 mg/dl	-0.02	0.17
Glucose CV	0.07	0.09
